# Defects of the Lamina Cribrosa in High Myopia and Glaucoma

**DOI:** 10.1371/journal.pone.0137909

**Published:** 2015-09-14

**Authors:** Atsuya Miki, Yasushi Ikuno, Tomoko Asai, Shinich Usui, Kohji Nishida

**Affiliations:** Department of Ophthalmology, Osaka University Graduate School of Medicine, Osaka, Japan; Purdue University, UNITED STATES

## Abstract

**Purpose:**

We evaluated the prevalence and characteristics of the defects of the lamina cribrosa (LC) in high myopia and glaucoma, and compared them with control eyes using swept-source optical coherence tomography (SS-OCT).

**Methods:**

One hundred fifty-nine eyes of 108 participants were divided into four subgroups; high myopia with glaucoma (MG, 67 eyes of 46 subjects), glaucoma without high myopia (G, 22 eyes of 13 subjects), high myopia without glaucoma (M, 35 eyes of 29 subjects), and a control group with neither glaucoma nor high myopia (C, 35 eyes of 20 subjects). The LC defects were identified and located using a standardized protocol in serial horizontal OCT scans. The prevalence rates of the defects were compared among the groups. Demographic and ocular factors were compared between eyes with and without defects.

**Results:**

LC defects were observed in one eye (0.03%) in the C group, 8 eyes (22.9%) in the M group, 11 eyes (50%) in the G group, and 28 eyes (41.8%) in the MG group. The prevalence rates of the defects differed significantly among the groups (P = 0.0009). Most eyes with defects in the G and MG groups (79.5%) had damage in the corresponding visual hemifields. Other factors such as visual acuity, intraocular pressure, axial length, refractive error, disc ovality, or parapapillary atrophy area did not differ significantly between eyes with and without LC defects.

**Conclusions:**

High myopia and glaucoma significantly increased the risk of LC damage. The LC damage in non-glaucomatous highly myopic eyes may at least partly explain the increased risk of developing glaucoma in myopic eyes.

## Introduction

Various experimental and histologic studies have shown that the optic nerve head (ONH) and the lamina cribrosa (LC) are the primary sites of axonal insults in glaucoma. For example, experimental studies have reported blockage of both orthograde and retrograde axonal transport by acute intraocular pressure (IOP) elevations at the level of the LC,[1,2] and consequent degeneration of cell bodies in some proportion of the retinal ganglion cells in animal glaucoma models.[3] In addition to transient axonal transport blockage, histologic studies have reported permanent distortion, compression, and posterior movement of the LC sheets.[4,5] Experimental studies also have identified the morphologic abnormalities of the LC such as posterior deformation, thickening, and hypercompliance in eyes of model animals with elevated IOP.[6,7] These studies support the notion that pressure-induced distortion of the LC impairs the trophic support to ganglion cell axons that leads to retinal ganglion cell death in glaucoma.

Recent advances in imaging technology, especially optical coherence tomography (OCT), have facilitated visualization of the morphologic characteristics of the LC in vivo.[8,9] Several earlier histologic and experimental studies of the glaucomatous LC abnormalities have been corroborated by recent in vivo imaging studies. These characteristics include thinning,[10] posterior deformation,[11] and focal defects of the LC in glaucomatous eyes.[12–15] Of these, focal LC defects were observed in 75% of glaucomatous eyes but rarely in healthy eyes. [12,14] Moreover, the location of the defects corresponded well with typical glaucomatous findings such as visual field defects, neuroretinal rim thinning, and retinal nerve fiber defects.[12,14,15] Another recent study reported a correlation between LC defects and visual field progression.[16] Together, focal defects of the LC may be considered as a clinical sign of the pressure-induced damage to the LC and ONH in glaucoma.

Myopia has long been recognized as a risk factor for glaucoma. Although a number of epidemiologic studies have shown a higher prevalence of glaucoma in myopic eyes,[17–19] the mechanism underlying the increased susceptibility of myopic subjects to glaucoma has not been fully elucidated. One possible link between myopia and glaucoma is increased stress acting on the LC due to elongation of the axial length. Cahane and Bartov theorized that myopic eyes have higher scleral tension across the LC than eyes with a shorter axial length, even when the IOP is the same.[20] The stress could be even greater in eyes with scleral thinning, which is a common morphologic feature of myopic eyes. Increased stress on the LC in myopic eyes may in turn lead to mechanical failure and deformation of the LC, such as the acquired pits of the ONH and peripapillary regions in highly myopic eyes reported by Ohno-Matsui and associates. [21]

In the current study using a swept-source (SS) OCT system, we evaluated and compared the prevalence rates and characteristics of the LC defects in four subgroups: high myopia with glaucoma, glaucoma without high myopia, high myopia without glaucoma, and healthy eyes with neither high myopia nor glaucoma. SS-OCT uses a long-wavelength light source of 1 micron (near infrared light) that can penetrate more deeply into tissue compared with the short-wavelength light used in conventional spectral-domain OCT devices. [22,23] With its ability to penetrate into deeper tissues, SS-OCT is suitable for imaging the LC. The aim of the current study was to test the hypothesis that mechanical damages of the LC are among key components in the link between myopia and glaucoma.

## Methods

### Study Population

This study included consecutive cases with glaucoma and/or high myopia who underwent SS-OCT imaging at the Department of Ophthalmology, Osaka University Hospital. Age-matched healthy control subjects were recruited from among the staff and employees of the hospital based on the following criteria; an IOP of 21 mmHg or lower, normal appearance of the ONH on fundus examination, and axial length between 22 and 26 mm. The eyes were categorized into four subgroups; high myopia with glaucoma (MG group), glaucoma without high myopia (G group), high myopia without glaucoma (M group), and age-matched healthy control eyes (C group). The diagnosis of glaucoma was based on the presence of glaucomatous optic neuropathy (localized or diffuse neuroretinal rim thinning and/or retinal nerve fiber layer defect) and an associated visual field defect. Visual fields were considered abnormal if (1) a glaucoma hemifield test value was outside the normal limits; and (2) at least 3 vertical, horizontal or diagonal contiguous test points in the same hemifield on the pattern deviation probability plot at P<5%, with at least 1 point at P<1%, excluding points directly above or below the blind spot; or (3) a pattern standard deviation of less than 5% of the normal reference values. High myopia was defined as having a refractive error of -6 diopter or greater, or an axial length of 26.5 mm or longer. Exclusion criteria included the presence of any other ocular or neurologic disorder that could cause visual field defects, a gonioscopically closed angle, secondary causes of IOP elevation, an anterior segment disorder or media opacity that affected image quality, and a history of intraocular surgery except uncomplicated cataract surgery.

All subjects underwent comprehensive ophthalmic examinations including measurement of the spherical equivalent refractive error using an auto refractometer (Nidek, Gamagori, Japan), measurement of the best-corrected visual acuity, Goldmann applanation tonometry, slit-lamp biomicroscopy, gonioscopy, dilated fundus examination, color photography of the optic disc (TRC-50DX, Topcon, Tokyo, Japan), and OCT. All subjects in the MG and G groups underwent SITA standard 30–2 visual field tests (Humphrey Field Analyzer; Carl Zeiss Meditec, Dublin, CA). All subjects with focal LC defects in the M and C groups underwent visual field tests performed by technicians masked to the clinical characteristics of the subjects to confirm the absence of an abnormal visual field.

The protocol of this study was approved by the institutional review board of Osaka University Hospital. Each participant provided written informed consent after explanation of the nature and possible consequences of the study. All study procedures adhered to the tenets of the Declaration of Helsinki for research involving human subjects.

### Swept-source OCT

A commercially available SS-OCT device (DRI-OCT, Topcon, Tokyo, Japan) was used to image the ONH and the parapapillary area. This device is based on SS-OCT technology, with a scanning speed of 100,000 A-scans/second. The center wavelength of the probe beam was 1,060 nm. Eyes were imaged using the 3D scan mode. With this scan protocol, 256 serial horizontal scans were acquired over a 6 x 6-mm cube centered on the ONH. Four images were taken in the same location and averaged to improve the image quality. Poor quality images such as poor contrast images due to media opacity, or poorly fixated images were excluded. Eyes with poor visibility of the LC, defined as less than 80% visibility of the anterior laminar surface within the ONH area also were excluded from analysis.

**Fig 1:**
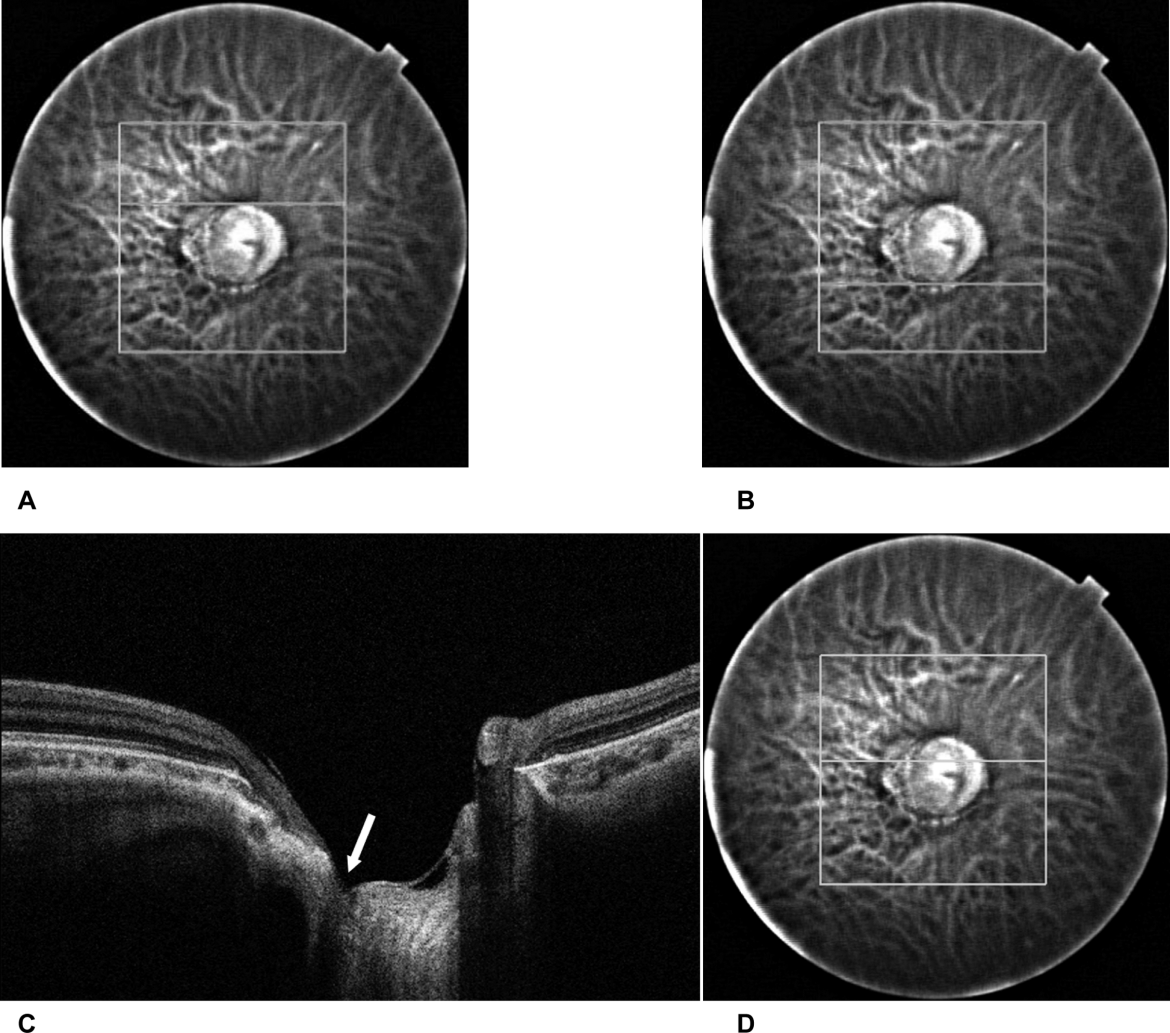
Identification of the defect location. A) The superior disc edge identified on a fundus image obtained with the SS-OCT in reference to the color fundus photograph. In this case, the superior disc edge was located at the 90th scan of the sequential horizontal scans. B) The inferior disc edge was identified in a way similar to the superior edge. In this case, the inferior disc edge was located at the 180th scan. This means the center of the disc is located at the 135th scan. C) The location of the defect. A defect of the lamina cribrosa was identified in the OCT image (arrow). In this case the defect was located from 115th through 118th scan, which means the defect location is in the superior half of the disc. D) In the reference fundus image, a horizontal white line shows the location of the scan.

A focal LC defect was defined as an anterior laminar surface irregularity violating the normal smooth contour based on previous reports.[12,14] To avoid false positives, defects needed to be >100 μm in diameter and >30 μm in depth, and also be present in 2 neighboring scans. The defect was located in either the superior or inferior half of the ONH as follows (Fig 1). First, the superior and inferior disc edges were identified in fundus images taken with the SS-OCT in reference to the color fundus photograph. The ONH then was divided into superior and inferior halves by the vertical center determined as the midpoint of the superior and inferior edges. The horizontal locations of the defects also were evaluated. Defects were located either temporal or nasal based on the location of the center of the defect.

### Corresponding visual field damage

We conducted a subgroup analysis of the correspondence between the locations of the LC defects and the corresponding visual hemifield damage in the MG and G group. The corresponding hemifield means the inferior hemifield in eyes that have LC defects in the superior half of the ONH, and superior hemifield in eyes that have LC defects in the inferior half of the ONH. We performed this analysis only in the MG and G groups, because by definition the eyes in the M and C groups had no visual field abnormality. In eyes in which the center of the largest LC break was in the superior half of the ONH, we assessed if the corresponding (inferior) hemifield was abnormal or not. In eyes with the largest LC break in the inferior half, we assessed the superior hemifield abnormality. In this analysis, a visual field abnormality in the hemifield was defined as three or more clustered non-edge abnormal test points in the pattern deviation probability plot with at least one point of p<1% in that hemifield.

### Disc parameters

**Fig 2:**
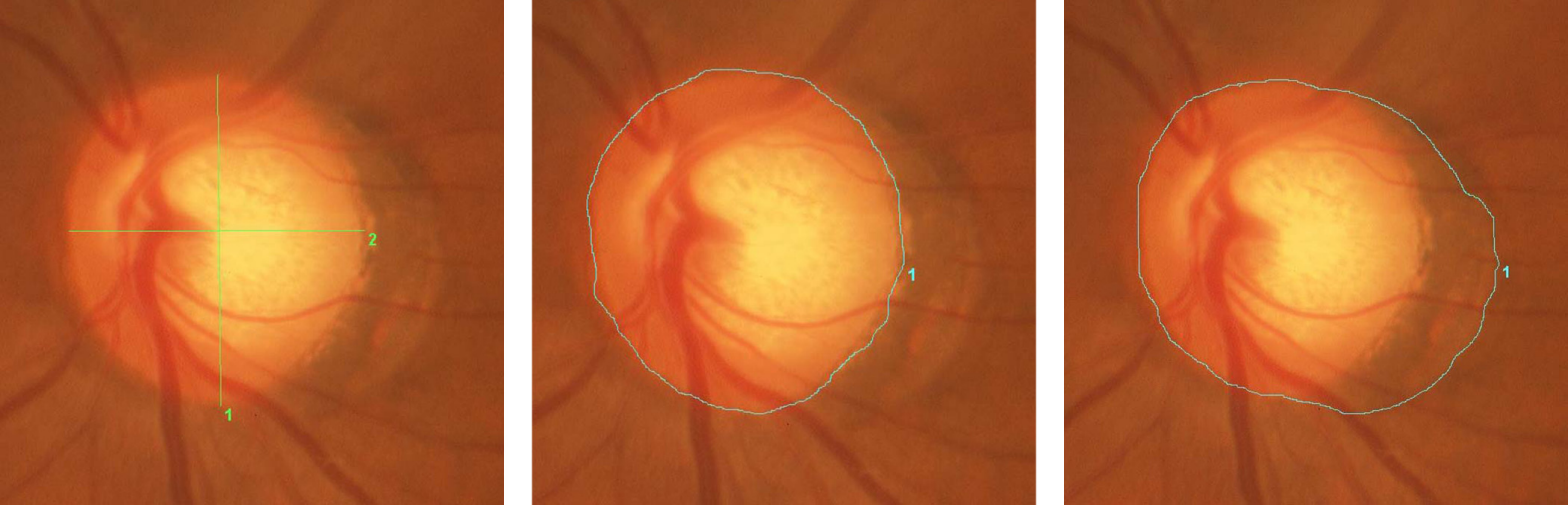
Measurement of disc parameters. Before the measurement, refraction, corneal curvature, and axial length were entered to the software to adjust for the ocular magnification effect. A) Ovality Index: Longest (1) and shortest (2) diameters of the optic disc were identified and manually traced. The length of the line was automatically calculated with the data management software (Imagenet R4). The ovality index was calculated by dividing the shortest disc diameter by the longest diameter. B) Disc Area: The edge of the optic disc was manually traced. The area surrounded by the line (= disc area) was automatically calculated. C) β-zone parapapillary atrophy area: First the line was manually drawn to enclose both optic disc and parapapillary atrophy. The area of the optic disc plus β-zone parapapillary atrophy was automatically calculated. Then the area of β-zone parapapillary atrophy was calculated by subtracting the disc area.

We evaluated the ovality index, disc area, and β-zone parapapillary atrophy (PPA) area as disc parameters. The ovality index is an index calculated by dividing the shortest disc diameter by the longest diameter as previously described.[24] For this analysis, digital color fundus photographs were imported into the IMAGEnet R4 Viewer image management system (Topcon). An investigator masked to clinical information manually delineated the shortest and the longest diameters, disc area, and β-zone PPA area. The parameters were calculated using the built-in measurement tools, while correcting for ocular magnification effect. In this system, the patient’s axial length, corneal radius, and refractive power were input into the Gullstrand schematic eye to calculate compensated values of length per pixel. (Fig 2).

### Statistical analysis

All statistical analyses were performed using commercially available software Stata version 12 (StataCorp, College Station, Texas). P values <0.05 were considered statistically significant. The baseline characteristics and the prevalence of the defects were compared statistically among subgroups. The demographic and ocular factors were compared between eyes with and without LC defects to evaluate the impact of the defects on those factors. Eye-specific factors were compared using mixed-effects modeling for numerical variables and clustered chi-square test for categorical variables to deal with possible inter-eye correlation within one subject. Subject-specific demographic factors were compared using one-way analysis of variance for numerical variables and chi-square test for categorical variables. For spherical equivalent refractive error, only phakic eyes were used for comparison.

## Results

Initially, a total of 222 eyes from 161 subjects met the inclusion criteria. Of those, 63 eyes of 53 cases were excluded because of poor visibility of the LC. As a result, 159 eyes of 108 subjects were included in the analysis. Sixty-seven eyes of 46 subjects were categorized in the MG group, 22 eyes of 13 subjects in the G group, 35 eyes of 29 subjects in the M group, and 35 eyes of 20 subjects in the C group. The baseline characteristics of the four subgroups are summarized in Table 1 (individual participants’ data are presented in S1 Table). Eighty-one eyes of 54 patients had normal tension glaucoma and 8 eyes of 5 patients had primary open angle glaucoma. Twenty-five eyes of 16 subjects (9 eyes in the C group, 1 eye in the G group, 3 eyes in the M group, and 12 eyes in the MG group) were pseudophakic and others were phakic. No significant differences in age or gender were seen among the groups. There was a marginally significant difference in the IOP between groups (P = 0.0176, mixed-effects model). Eyes in the MG and the M groups had significantly myopic refractive error and longer axial length compared with the C group (P<0.0001). Visual field mean deviation of eyes in the G group and the MG group were -10.3 ± 7.9 dB and -10.5 ± 8.1 dB (average ± standard deviation), respectively.

**Table 1:** Baseline Demographics of the four groups.

	High myopia with glaucoma (MG group)	Glaucoma without high myopia (G group)	High myopia without glaucoma (M group)	Healthy control (C group)	Total	P-value
Eyes / Subjects	67 / 46	22 / 13	35 / 29	35 /20	159 / 108	
Age (years)	52.4 ± 12.4	62.3 ± 10.4	55.0 ± 13.5	57.4 ± 16.6	55.2 ± 13.5	0.1076
Gender (F/M)	32 / 14	10 / 3	21 / 8	17 / 3	80 / 28	0.609
IOP (mmHg)	15.0 ± 5.2	12.8 ± 2.2	16.3 ± 3.1	14.7 ± 2.5	14.9 ± 4.1	0.0176
Spherical equivalent (D)	-8.1 ± 3.7	-3.0 ± 2.4	-10.6 ± 5.5	-1.0 ± 1.5	-6.7 ± 5.1	<0.0001
Axial length (mm)	27.8 ± 1.5	24.5 ± 0.9	28.9 ± 1.7	23.5 ± 0.7	26.6 ± 2.5	<0.0001

F, female; M, male; IOP: intraocular pressure; D, diopters. P values represent the result of comparison between groups with mixed-effects modeling (numeric variables) or clustered chi-square test (categorical variables).

### Number and location of LC defects

**Fig 3:**
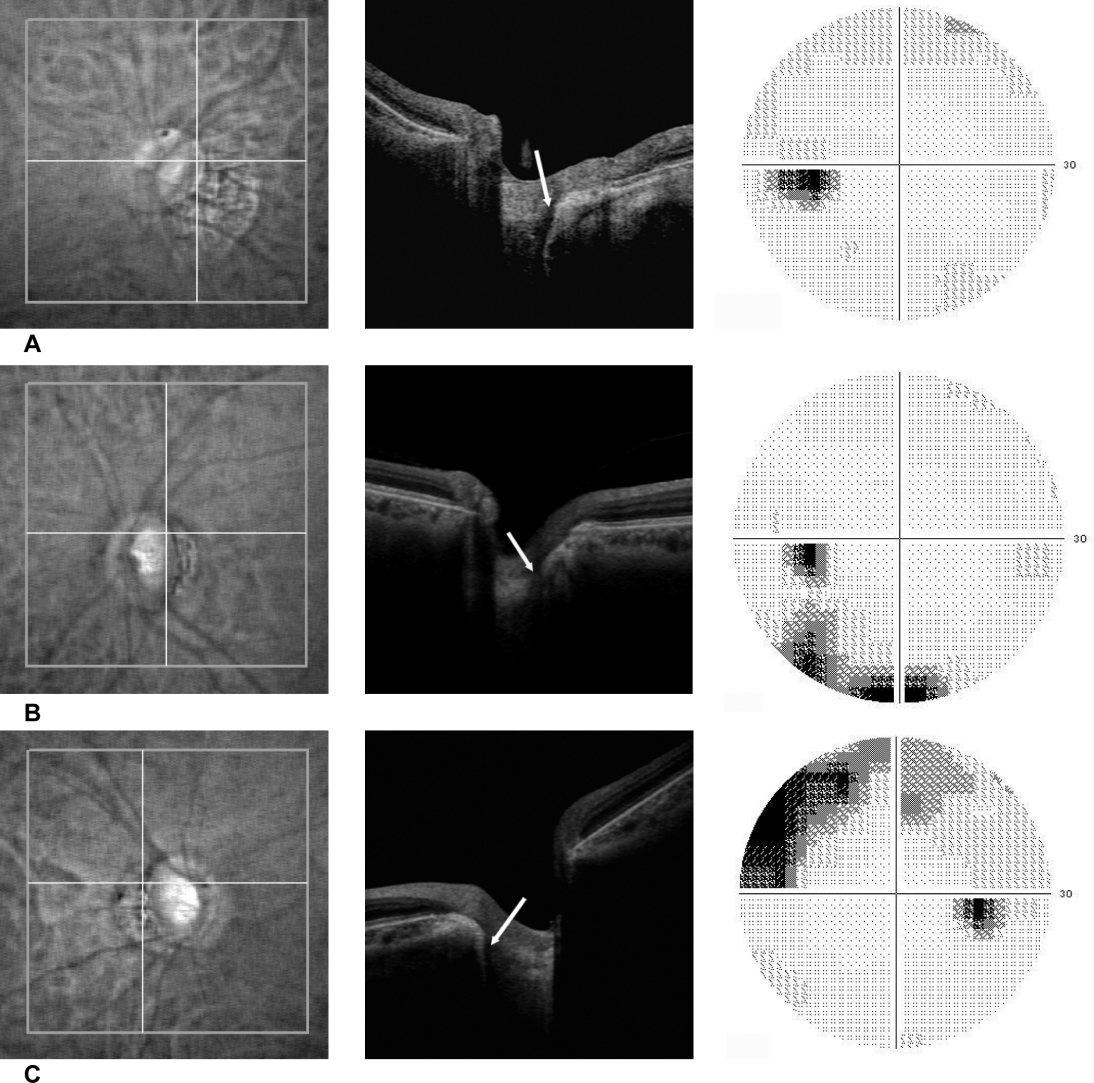
Representative cases with LC defects in A) the M group (high myopia without glaucoma), B) the G group (glaucoma without high myopia), and C) the MG group (glaucoma with high myopia), respectively. Each panel shows (from left to right) infrared fundus images, OCT images, and SAP visual field printouts. The locations of the scan lines are shown as horizontal lines in infrared images (left panels). The arrows in OCT images show the locations of the defects (center panels).

LC defects were found in 28 of 67 eyes (41.8%) in the MG group, 11 of 22 eyes in the G group (50.0%), 8 of 35 eyes (22.9%) in the M group, and only 1 of 35 eyes (0.03%) in the C group (Table 2). Representative cases with LC defects in the M, G, and MG groups are shown in Fig 3. The proportion of having at least one defect was significantly different among the groups (P = 0.0009, clustered chi-square test). A pair-wise comparison using the chi-square test with the Bonferroni correction showed significant differences between the G and M, G and C, and M and C groups (P = 0.002, 0.0006, and 0.0304, respectively). Forty-three eyes had only one defect, whereas 5 eyes had 2 defects (4 eyes in the MG group and 1 eye in the G group).

**Table 2:** Prevalence of LC defects in the four study groups.

	Total eyes in group	Eyes with LC defects
High myopia with glaucoma (MG)	67	28 (41.8%)
Glaucoma without high myopia (G)	22	11 (50%)
High myopia without glaucoma (M)	35	8 (22.9%)
Healthy control (C)	35	1 (0.03%)
Total	159	48 (30.2%)
P-value		0.0009

LC, lamina cribrosa

The center of the largest LC defect was in the superior half of the LC in 23 eyes (47.9%) and in the inferior half in 25 eyes (52.1%). The horizontal location of the largest LC defects was predominantly in the temporal half of the disc (47 eyes, 97.9%). The locations of the LC defects were different among disease types. The majority of the largest defects were located in the inferior half of the ONH in the G group (7/11 eyes, 63.6%), whereas the majority of the largest defects were located in the superior half in the MG group (15/28 eyes, 53.6%), and half of the largest defects were located in the superior half in the M groups (4/8 eyes, 50%). The difference was statistically significant (P = 0.0002, clustered chi-square test).

### Corresponding visual field damage

Of the 39 eyes with LC defects in the MG and G groups, a reliable visual field test was obtained in all eyes. Nineteen eyes had the largest LC defects in the superior half of the ONH, and 20 eyes had the largest LC defects in the inferior half. The inferior hemifield of eyes that had superior LC defects was abnormal in 14 of 19 eyes (73.7%). The superior hemifield of eyes that have inferior LC defects was abnormal in 17 of 20 eyes (85.0%). Overall, the corresponding visual hemifield was abnormal in 31 of 39 eyes (79.5%).

### Comparison between eyes with and without LC defects

There was no significant difference between eyes with and without LC defects regarding age, gender, refractive error, axial length, the ovality index, and the disc area in the univariate comparison (Table 3). There was a marginal difference in the mean β-zone PPA area between eyes with and without defects, but the difference did not reach significance.

**Table 3:** Demographic and Ocular Factors in Eyes with and without LC Defects.

	Eyes with LC defects	Eyes without LC defects	P-value
Gender (Female)	26 (76.5%)	54 (73.0%)	0.700
Age (years)	55.3 ± 11.9	55.2 ± 14.3	0.9722
Spherical equivalent (D)	-7.6 ± 3.7	-7.7 ± 5.7	0.9839
Axial length (mm)	27.1 ± 1.9	26.3 ± 2.6	0.5606
IOP (mmHg)	15.1 ± 4.3	14.9 ± 4.0	0.5687
Ovality Index	0.79 ± 0.13	0.83 ± 0.12	0.2738
Disc area (mm^2^)	3.07 ± 1.40	3.14 ± 1.16	0.6256
Beta-zone PPA area (mm^2^)	4.31 ± 4.49	3.17 ± 4.38	0.1484

LC, lamina cribrosa, D, diopters; IOP, intraocular pressure, PPA, parapapillary atrophy

P values represent the result of a comparison between eyes with and without defects with mixed-effects modeling (numeric variables) or clustered chi-square test (categorical variables).

### Representative cases

Representative cases with LC defects in the M, G, and MG groups are shown in Fig 3.

*M group*. A 58 year-old woman had high myopia (-12 dipopter) in the left eye. The optic disc was tilted, but both the superior and the inferior neuroretinal rim were intact. A focal defect of the LC was observed in the inferior temporal part of the disc in the OCT image. The visual field was within normal range (Fig 3A).

*G group*. A 66 year-old woman with normal tension glaucoma. The refractive error was -0.5 diopter. Optic disc photograph showed a thin superior, temporal, and inferior neuroretinal rim, and superior temporal retinal nerve bundle defect. An OCT image showed a focal defect in the superior temporal area of the LC. The visual field showed a corresponding inferior nasal scotoma (Fig 3B).

*MG group*. A 40-year man had primary open angle glaucoma and myopia (-11.5 diopter). A focal defect was observed in the superior temporal part of the LC. Visual field examination showed glaucomatous damage, but the field corresponding to the location of the LC defect was intact (Fig 3C).

## Discussion

The current study evaluated the prevalence and locations of defects in the LC in four subgroups; highly myopic eyes with glaucoma (MG group), glaucomatous eyes without high myopia (G group), highly myopic eyes without glaucoma (M group), and healthy control eyes without high myopia (C group). As in previous reports, LC defects were commonly found in glaucomatous eyes. In addition, LC defects were also observed in highly myopic eyes without any clinical sign of glaucomatous optic neuropathy. The prevalence of LC defects was 16.1% in highly myopic eyes without glaucoma, which was significantly lower than in highly myopic eyes with glaucoma (41.8%) or glaucomatous eyes without high myopia (50%), but much higher than in healthy eyes without high myopia (0.03%). Eyes with LC defects had larger area of the β-zone PPA compared to eyes without LC defects.

LC defects have been associated with glaucomatous optic neuropathy. In the current study, 79.5% of eyes with LC defects in the MG and G groups had corresponding visual field damage. This good structure-function relationship supports the notion that formation of LC defects plays a role in the pathology of glaucomatous optic neuropathy. A small proportion of eyes did not have visual field damage corresponding to the LC damage. One possible explanation for this apparent discrepancy is that the LC defects occur earlier than the visual field damage in the course of glaucomatous damage. Another possibility is that some of field damages corresponding to LC defects are too small or too subtle to be detected in standard visual field examinations. In addition, the current study showed for the first time that a considerable proportion of highly myopic eyes without any apparent sign of glaucomatous optic neuropathy also have LC defects. Highly myopic eyes exhibit characteristic appearance of the optic nerve head such as oval configuration, large disc area, and large area of PPA. [25,26] Therefore, we investigated the correlation between LC defects and disc parameters including the ovality index, disc area, and β-zone PPA area. The mean β-zone PPA area in eyes with LC defects was larger than in eyes without defects, but the difference did not reach significance. There was also no significant difference in the ovality index or disc area, and other demographic and ocular factors such as age, gender, refractive error, and axial length between eyes with and without LC defects.

It is also unclear whether LC defects in myopic eyes are pathologically related to glaucoma or not. The first hypothesis is that LC defects can occur in myopic eyes independently of glaucoma. The morphologic characteristics of the ONH in myopic eyes such as large disc area, oval configuration, and large PPA may be regarded as evidence of increased stress on the ONH in highly myopic eyes, as supported by the results of a mathematical modeling study. [20] A recent report about the acquired pit of the ONH in myopic eyes further supports this hypothesis. [21] However, as mentioned earlier in this manuscript, we did not find clear evidence of a correlation between LC defects and other characteristics of ONH morphology in myopic eyes. The LC defects in myopic eyes also may be a sign of glaucomatous optic nerve damage occurring in the very early stage when optic neuropathy cannot be detected with existing clinical examinations. This hypothesis agrees with the study of Bellezza and associates who reported that plastic and hypercompliant deformation of the LC occurred in the early stage of experimental glaucoma. [6] In addition, the prevalence of LC defects did not differ significantly between the G and MG groups, which may mean that the existence of myopia does not further increase damage of the LC in established glaucoma. This also agrees with the hypothesis that LC defects in myopia are an early sign of glaucoma. However, longitudinal follow-up of eyes with LC defects in the M group is necessary to answer this question.

In the current study, the largest LC defects were in the superior half of the ONH in 23 (47.9%) eyes and in the inferior half in 25 (52.1%) eyes. In previous investigations, defects were found dominantly in the inferior area of the ONH. Tatham and associates reported that 19 eyes had inferior LC defects, whereas only 2 eyes had superior LC defects. [14] Kiumehr et al. reported that 67 defects were in the inferior area and 31 defects were in the superior area.[12] Preferential laminar damage in the inferior half of the ONH in these studies agreed with the fact that the superior visual field is more susceptible to glaucomatous damage in the early stage of the disease. [27] In this study, we did not see such selective occurrence of defects in the inferior portion, possibly because the majority of our participants were highly myopic, in contrast to previous reports that were not focused on high myopia. In fact, the current results showed a possible correlation between the location of the LC defect and myopia. In the G group, the LC defects were in the inferior half of the ONH in 63.6% of the eyes; in contrast, the LC defects were in the inferior half of the ONH in only 46.4% of the MG group. Deformation of the ONH due to high myopia may explain this inconsistency in defect locations between highly myopic and non-highly myopic eyes. Jonas and Dichtl reported that optic disc morphology in glaucomatous eyes differs significantly between highly myopic eyes and eyes without high myopia. [28] Greve and Furuno showed that atypical retinal nerve fiber layer defects were seen frequently in myopic glaucomatous eyes but rarely in non-myopic glaucomatous eyes. [29] Further research on the locations of LC defects in myopia and glaucoma may improve our understanding of these pathologies.

The current study had several limitations, including the hospital-based design, relatively small sample size, and the marginal difference in age and IOP among the subgroups. A larger scale, prospective, longitudinal study would more clearly show risk factors and the pathological significance of the LC defects in glaucoma and myopia. Nevertheless, this is the first report to show the increased risk of having LC defects in highly myopic eyes, and about the possible relationship between the LC damage and PPA. These findings would help us gain a better understanding of the pathophysiology of high myopia and glaucoma.

In conclusion, focal LC defects were found in glaucomatous eyes and highly myopic eyes without glaucoma. The prevalence of defects was the highest in glaucomatous eyes without high myopia (50%) and highly myopic glaucomatous eyes (41.8%), followed by highly myopic eyes without glaucoma (22.9%) and control eyes (0.03%). Most glaucomatous eyes with LC defects (75.9%) had visual field defects in the corresponding hemifield. The locations of the defects differed between eyes with and without high myopia. The increased risk of having LC defects in highly myopic eyes compared with control eyes may be related to the higher susceptibility of highly myopic eyes to glaucoma. The current results suggest the importance of evaluating the LC in glaucomatous eyes as well as in myopic eyes.

## Supporting Information

S1 Table Baseline demographics of the whole participants. (XLSX)
